# Measurement of Mental Workload in Clinical Medicine: A Review Study

**DOI:** 10.5812/kowsar.22287523.2045

**Published:** 2011-09-26

**Authors:** Aidan Byrne

**Affiliations:** 1 Clinical Skills and Simulation, School of Medicine, Cardiff University, Cardiff, UK

**Keywords:** Workload, Patient simulation, Medical errors

## Abstract

**Background:**

Measures of mental workload are now commonly used in industries to identify sources of error and to improve performance.

**Objectives:**

This study aimed to review the evidence for the use of this technique within medicine.

**Patients and Methods:**

We used search engines and the internet to identify experimental studies that included a measure of mental workload in medical practitioners or trainees/students. Studies that aimed to measure mental “stress” as a disorder, or “productivity” were excluded. Each abstract and then the full paper were appraised prior to inclusion.

**Results:**

Thirty-three studies were identified that matched the inclusion criteria. Although these covered a variety of settings, common methods were identifiable. The results support the concept of mental workload measurement as an important factor in medical performance.

**Conclusions:**

The limited number of studies and the variety of definitions and measurement techniques used in these studies, make direct comparisons difficult. However, the utility of this methodology in medical education appears to have been established, and guidelines for further research methods are proposed.

## 1. Introduction

**Figure 1.**
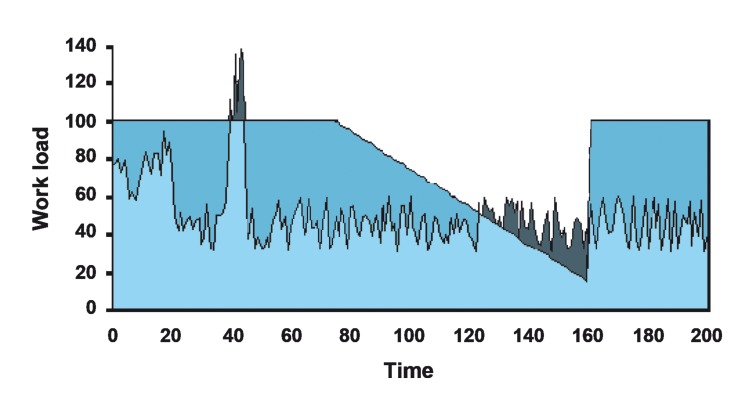
Illustration of How Mental Workload May Vary During an Anesthesia

It is generally accepted that there are limits on human ability to process information, and that information overload can lead to poor performance (1). For example, many people will have experienced the difficulties of simultaneously trying to drive, navigate, read road signs, and listen to passengers (2). Although no widely accepted definition of mental workload exists, it can be seen as an interaction between the demands of the task and the performance of the operator (3). Most researchers regard the maximum rate at which an individual can process information as their workload capacity, and the amount of this capacity in use at any one time as their mental workload. Figure 1 is an illustration of how the mental workload of an anesthetist may vary during the administration of an anesthetic. Time is shown on the x-axis and total workload is shown on the y-axis, with the mental capacity (up to 100%) and the mental workload. During the first 20 minutes of anesthesia induction, workload is high, but within capacity. Thereafter, during maintenance, the workload falls to a lower level. At 35 minutes, a crisis leads to a sudden increase in workload, which exceeds capacity. This sudden overload is a common feature of critical incidents and is known in aviation as “maxing out. ” Information that was not processed is also shown in the figure 1. Once the crisis has passed, workload rapidly falls below capacity. However, at around 80 minutes, the anesthetist becomes progressively more tired and his/her capacity gradually falls. Again, between 120 and 160 minutes, workload exceeds capacity and information is not processed. At 160 minutes, for whatever reason, the anesthetist is roused, capacity increases, and the task is completed within workload.

Although excessive mental workload has been identified in many fields, it does not necessarily imply poor performance. For example, having a telephone conversation while driving is inadvisable because we may not have the capacity to engage in conversation and drive at the same time. However, this does not mean that using a telephone while driving will lead to an accident; it just makes an accident more likely. In the same way, a doctor who is overloaded with information, or who has impaired ability, may practice safely, but be more prone to making errors (4).

Using this model, the mental workload of a doctor can be measured in four ways:

1. Primary task intensity, (number of cases seen, knots tied)
2. Objective physiological measures, (heart rate or skin conductivity)
3. Objective psychological measures (secondary tasks)
4. Subjective operator measures (questionnaires)

For example, the mental workload of a doctor in an outpatient clinic could be estimated from the number of patients seen or the number of procedures performed (primary task). Similarly, increased mental workload would also be likely to be associated with increased heart rate or decreased skin conductivity. Secondary tasks involve an additional simple task such as responding to a light or bleep, or performing mental maths, that subjects are asked to complete in addition to their primary task. If the subjects’ mental workload exceeds their capacity, performance of the secondary task will deteriorate. Therefore, if the performance of the secondary task is monitored, deterioration suggests excessive mental workload. Subjective workload is conventionally measured with a questionnaire asking subjects to rate the difficulty of the task, usually with a series of questions aimed at, for example, physical workload, frustration, perceived success, and cognitive effort.

In simple terms, for an inexperienced doctor at a busy clinic, the number of patients seen would be high (primary task), the doctor’s heart rate would be increased (physiological measure), telephone calls would be ignored (secondary task), and the experience would be rated as difficult (subjective measure). On the other hand, if the inexperienced doctor were replaced by an experienced practitioner, it is likely that their heart rate would increase less, calls could be answered more often, and the experience would be rated as less stressful. The principles of such measurements (1, 3, 5), as well as their specific application to the medical environment (4, 6, 7) have been described elsewhere. It is widely accepted that multiple measures are required, because workload has a complicated relationship with both ability and effort. For example, a poorly performing doctor in the above situation might rate the consultation as easy, have an unchanged heart rate, and be able to answer telephones (suggesting high levels of performance). However, this apparent high level of performance might be due to their complete inability to interact successfully with patients. The challenge for researchers in this field is to design valid and reliable instruments for the measurement of mental capacity and workload (6, 8).

## 2. Published Evidence

A wide variety of medical specialities have used these techniques, including anesthesia (9-23), surgery (24-29), general medicine (30, 31), emergency medicine (32, 33), intensive care (34), radiology (35), ward staff (36, 37), primary care physicians (31, 38), and medical students (39).

The settings for the studies were also diverse, including simulators (9-14, 17, 19, 24-29, 34, 36, 37, 39), operating theatres (15, 16, 18, 20-23), outpatient/ambulatory care centres (30, 31, 38), emergency rooms (32, 33), general wards (36, 37), and radiology reporting rooms (35). However, most were small-scale studies with an average of 29 subjects (range, 9–116), and an average of 158 procedures (range, 9–2053). The workload associated with the primary task was defined by non-standardized measures such as task analysis (20, 22), number of patients seen (30, 31), or number of knots tied (24, 25). Other studies used an “unloaded” period to compare to the main task studied (12, 39). The use of widely accepted and validated measures of primary task workload would make comparison between studies easier; however, such measures were used in a minority of the studies (26, 27). The methods used to measure the primary task workload were equally diverse and used data from simulators (14), rating of videotapes by trained observers (11, 35), observed counts of procedures completed (11), clinical record-keeping (10), reaction times (9), time-in-motion studies (16), and the number of observed errors (25). An objective measure of workload was used in 14 studies and also varied widely in method. These included time taken for the subject to respond to a change in a visual stimulus (16, 20-22, 26), heart rate derived from the electrocardiogram (22), accuracy of 2-number mental arithmetic (15), response time to a tactile stimulus (12, 39), accuracy of the clinical record (10), skin conductance (33), and eye-blink rate (24).

Subjective workload was measured with either the NASA TLX form (9, 11, 14, 17, 19, 25, 26, 28, 32-37), the Borg workload score (20-22), or other paper-based, unvalidated forms (13, 15, 23, 24, 38). The conclusions of these studies suggest that mental workload is reduced by using speech-input records compared to written records (13), with experience (15, 21, 23, 26, 27, 29, 30), using a mixed graphical-numeric interface (11), using drug administration devices that provide feedback (14), with an improved electronic interface (17, 37), with the addition of instruction to training, (25) with increased practice (28), and with digital rather than hard-copy x-rays (35). Workload was increased with fatigue (30, 31), number of patients seen (30, 31, 38), dissatisfaction (30, 31), poor self-rating of performance (30, 31), poor observer rating of performance (30), laparoscopic compared with open surgery (24), during an anesthetic crisis (10), during induction of anesthesia (15, 16, 20-22), with more difficult cases (15, 23, 26, 38), with increased administrative tasks (38), when students are present (22), and when using transesophageal echo during anesthesia (20).

## 3. Practical Considerations

The relevance of mental workload to performance has already been identified, both as a theoretical possibility and as part of published studies (4, 6, 7, 26). It shares many concepts with Cognitive Load Theory (40), which emphasizes the need to recognize the limited cognitive abilities of learners when designing educational processes. The principles guiding the measurement of mental workload are well established and there is a very wide range of literature describing such studies in non-medical environments (1, 3, 5, 6). For example, the effects of using a mobile phone while driving have been measured using mental workload techniques (41).

It is also recommended that multiple measures be used, and that primary workload be included. This is both because currently available methods are not adequate to use in isolation, and because workload can vary in unexpected ways (1, 6, 8). For example, in a previous study, the heart rate of a subject was found to be raised during the “normal” phase of a simulation (in anticipation of a problem) only to fall toward normal during the simulated problem (42), presumably because the problem was not as bad as anticipated. In the same way, the mental workload of novices may be lower than more experienced trainees because they have yet to appreciate the difficulties facing them; this is termed “unconscious incompetence” (43). The principle difficulty faced by researchers is the establishment of standardized measures of mental workload and their normal ranges so that valid comparisons can be made between subject groups. Primary task measures are relatively easy to define in procedural skills, for example, the number of knots tied per minute by a laparoscopic trainer. However, it may also be possible to define a range, for example, of history-taking tasks with defined levels of complexity, in the same way that the workload associated with a variety of airway procedures has already been established (23). Objective measures of workload are more difficult to define, as theoretical approaches to the problem emphasize that workload has multiple aspects that may be measured separately (1). These aspects are often linked to specific neurological processes. For example, it is recognized that it is possible to a watch a monitor (visual task) while also listening to a conversation (auditory task). It is also possible to watch a monitor (sensory) and run through possible diagnoses (cognitive). However, it is difficult or impossible to listen to two conversations (auditory-auditory), watch two different monitors (visual-visual), or run through diagnoses and calculate a drug dose at the same time (cognitive-cognitive).

It is therefore crucial that the objective measure chosen is appropriate to the task chosen. For example, subjects asked to calculate drug dosages (cognitive), may not show any change in reaction time to a warning light (visual), because different resources are being used. More appropriate measures, for example, would be the response to a pattern stimulus (visual) while performing laparoscopic surgery (visuo-motor), (27) or response to a tactile stimulus (monitoring) during anesthesia (monitoring) (12). Physiological measures such as heart rate have fewer problems in that they are less resource-specific. For example, subjects are likely to sweat more whether overloaded by visual, auditory, or cognitive tasks. However, these tasks may themselves be more intrusive and subject to physical effects (21, 22). For example, the increased heart rate of a subject performing chest compression during resuscitation is unlikely to be entirely due to increased mental workload. Further, the complex medical environment makes it inappropriate to directly transfer techniques used in other environments such as aviation, for example, as pilots work in a standardized, constrained environment where monitors may be placed in fixed locations. In contrast, even in anesthesia, which is often compared to aviation, staff move between rooms and often use a variety of equipment in different settings (15). Subjective measurements are less complex in that a simple questionnaire can be used. The NASA TLX (44) questionnaire has been validated in other areas and is freely available for non-commercial use. The Borg Workload Scale (45) has also been used in a minority of studies, but is less well validated. Clinicians may feel that the reduction of clinical practice to a set of numbers is inappropriate. We agree with others (46) that expertise alone is not the hallmark of a competent doctor but “rather the manner in which individuals choose to approach their work. ” Measured mental workload is only one aspect of performance; however, it may provide vital insights into ways to make medicine safer. Recent research has already linked measured mental workload with clinical outcomes (47).

## 4. Conclusions

Mental workload is a concept that may be used as a method of assessment, to determine the effect of training, and perhaps also as a component of performance assessment. Further studies should include, as a minimum, a measure of the primary workload, an objective measure of workload, and a measure of subjective workload. Studies should avoid the use of new methods that have not yet been validated, unless used in addition to an established method for comparative purposes. In particular, subjective workload should use the NASA TLX score as this has been widely validated in other fields and has been used in the majority of studies reviewed in this paper. Whenever possible, additional techniques should be included so that comparisons between measurement techniques can be made. For example, in a study of primary task workload in an outpatient department, primary workload could be measured in terms of the number of patients seen, case difficulty rating, and observer rating.

It must also be recognized that mental workload should be evaluated as a single aspect of medical performance, and not confused with the concepts of competence or effective practice.
